# Phenotype of CNTNAP1: a study of patients demonstrating a specific severe congenital hypomyelinating neuropathy with survival beyond infancy

**DOI:** 10.1038/s41431-018-0110-x

**Published:** 2018-03-06

**Authors:** KJ Low, K Stals, R Caswell, M Wakeling, J Clayton-Smith, A Donaldson, N Foulds, A Norman, M Splitt, K Urankar, K Vijayakumar, A Majumdar, DDD Study, S Ellard, SF Smithson

**Affiliations:** 1 Department of Clinical Genetics, St Michaels Hospital, Bristol, UK; 2 School of Clinical Sciences, University of Bristol, Bristol, UK; 3 Department of Molecular Genetics, Royal Devon & Exeter NHS Foundation Trust, Exeter, UK; 4 Institute for Biomedical and Clinical Science, University of Exeter Medical School, Exeter, UK; 5 Manchester Centre for Genomic Medicine, St Marys’ Hospital, Manchester, UK; 6 Institute of Human Development, University of Manchester, Manchester, UK; 7 Wessex Clinical Genetics Service, Southampton, UK; 8 Northern Genetics Service, Institute of Genetics Medicine, Newcastle upon Tyne, UK; 9 Department of Neuropathology, North Bristol NHS Trust, Bristol, UK; 10 Paediatric Neuromuscular Service, Bristol Royal Hospital for Children, Bristol, UK; 11 Wellcome Trust Sanger Institute, Cambridge, UK

## Abstract

CHN is genetically heterogeneous and its genetic basis is difficult to determine on features alone. *CNTNAP1* encodes CASPR, integral in the paranodal junction high molecular mass complex. Nineteen individuals with biallelic variants have been described in association with severe congenital hypomyelinating neuropathy, respiratory compromise, profound intellectual disability and death within the first year. We report 7 additional patients ascertained through exome sequencing. We identified 9 novel *CNTNAP1* variants in 6 families: three missense variants, four nonsense variants, one frameshift variant and one splice site variant. Significant polyhydramnios occurred in 6/7 pregnancies. Severe respiratory compromise was seen in 6/7 (tracheostomy in 5). A complex neurological phenotype was seen in all patients who had marked brain hypomyelination/demyelination and profound developmental delay. Additional neurological findings included cranial nerve compromise: orobulbar dysfunction in 5/7, facial nerve weakness in 4/7 and vocal cord paresis in 5/7. Dystonia occurred in 2/7 patients and limb contractures in 5/7. All had severe gastroesophageal reflux, and a gastrostomy was required in 5/7. In contrast to most previous reports, only one patient died in the first year of life. Protein modelling was performed for all detected *CNTNAP1* variants. We propose a genotype–phenotype correlation, whereby hypomorphic missense variants partially ameliorate the phenotype, prolonging survival. This study suggests that biallelic variants in *CNTNAP1* cause a distinct recognisable syndrome, which is not caused by other genes associated with CHN. Neonates presenting with this phenotype will benefit from early genetic definition to inform clinical management and enable essential genetic counselling for their families.

## INTRODUCTION

Nerve fibres are myelinated in segments to enable high velocity conduction. This myelination occurs through the wrapping of glial cells around the nerve fibre in between the nodes of Ranvier [1]. The nodes of Ranvier are flanked by paranodal junctions and together they form distinct domains, which are crucial for saltatory conduction. These junctions are composed of multiple molecules, which include *Drosophila* Neurexin IV-related protein, Caspr/Paranodin, initially termed ncp1 [1]. Ncp1, now more commonly referred to as the rat contactin-associated protein, CASPR [2], forms part of a high molecular mass complex in the paranodal junction and interacts with contactin. In a homozygous null mouse model (ncp1-/ncp1-), ataxia, tremor, hypomotility, wide-based gait, significant motor paresis and severely decreased peripheral nerve conduction velocity was associated with disorganization of the paranodal junctions and dysregulation of ion channel interactions [1]. Death occurred within the first month of life. Heterozygous mice were unaffected at all ages. Immunohistochemical studies demonstrated the expression of caspr at the paranodal region in mouse sciatic nerve and the central nervous system. These findings suggest that the development of normal paranodal junctions is CASPR-dependent and its absence leads to disruption of the paranodal loops.

*CNTNAP1* (MIM # 602346) encodes CASPR and was first implicated in human disease in 2014 [3]. Using the whole-exome sequencing (WES) approach in 31 non-syndromic fetal hypomobility/arthrogryposis multiplex congenital families, Laquerriere et al. identified four unrelated families with homozygous truncating variants [3]. Death occurred within the first 40 days of life in all four cases. *CNTNAP1* variants have since been described in association with congenital hypomyelinating neuropathy (CHN) [4], which comprises prenatal-onset congenital neuropathy, areflexia, hypotonia, hypomyelination, slow nerve conduction velocities and arthrogryposis. Nizon et al. [5] described two brothers with severe congenital hypotonia, foot deformities and markedly decreased nerve conduction in association with compound heterozygous variants in *CNTNAP1*, including the first causative missense variant. A separate study investigated nerve biopsies of these brothers and an additional sibling-pair with CHN who had compound heterozygous nonsense/missense variants [6]. These patients had identical hypomyelinated nerves with characteristic lesions in the paranodal area where CASPR is located, which were reminiscent of those in the sciatic nerves of caspr-1 null mice. Mehta et al. [7] reported a neonate with CHN who had homozygous *CNTNAP1* missense variants that are predicted to affect function with typical clinical presentation, absent sensory nerve and compound muscle action potentials and hypomyelination on nerve biopsy, who died after withdrawal of life, sustaining care at 1 month of age. In a WES study of 71 patients with white matter abnormalities, Vanderver et al. [8] identified two further cases (one homozygous missense, one compound missense/nonsense), of *CNTNAP1-*associated CHN. Very recently, two further reports [9, 10] have been published describing further 8 cases, bringing the reported total to 19 (see Supplementary Table 3 for review of previously reported cases).

Here we present clinical and molecular data from seven additional patients with biallelic *CNTNAP1* variants identified via exome sequencing undertaken for undiagnosed developmental disorders and CHN. The clinical features we observed in these patients, and those previously published, suggest that there is a distinct *CNTNAP1*-related congenital hypomyelinating neuropathy syndrome which is not always lethal in the neonatal period.

## METHODS

### Patient ascertainment

Five of the affected patients were recruited via the Deciphering Developmental Disorders (DDD) study (www.ddduk.org) open to the UK NHS Regional Genetics Services (see Table 1for associated Decipher IDs) [11]. All five were the only affected cases in their families. Patient 2 and 3 (siblings) were recruited through their local Clinical Geneticist. All patients were assessed by their Clinical Geneticist who assisted with systematic detailed phenotyping. Patient growth centiles and *z* scores were calculated using UK WHO data (www.rcpch.ac.uk/growthcharts).

**Table 1:** Clinical and genetic findings in patients 1–7 and previously reported patients with CNTNAP1 variants

Individual	1	2	3	4	5	6	7	**Total (n = 7)**
Study ID	Decipher265308	EX1603954 (Decipher351768)	EX1603955 (Affected brother of Patient 2)	Decipher260496	Decipher272467	Decipher301316	Decipher285508	
cDNA (NM_003632.2) Intronic variants according to NG_042091.1	c.2141[G>C];[G>C]	c.[635T>C];[1677G>A]	c.[635T>C];[1677G>A]	c.2344[C>T];[C>T]	c.[1735+1G>A];[2344C>T]	c.[149C>A];[2600del]	c.[1861C>T];[2687G>A]	
Protein	p.[(Arg714Pro)];[(Arg714Pro)]	p.[(Leu212Pro)];[(Trp559Ter)]	p.[(Leu212Pro)];[(Trp559Ter)]	p.[(Arg782Ter)];[(Arg782Ter)]	p.[?]; [(Arg782Ter)]	p.[(Pro50Gln)];[(Asp867fs)]	p.[(Arg621Ter)];[(Trp896Ter)]	
Genomic (GRCh37)	chr17:g.[40843236 G>C]	chr17:g.[40837358T>C];[40841587G>A]	chr17:g.[40837358T>C];[40841587G>A]	chr17:g.[40843529 C>T]	chr17:g.[40841646 G>A];[40843529 C>T]	chr17:g.[40835920 C>A];[40844586del]	chr17:g.[40842762 C>T];[40844673 G>A]	
Zygosity	Homozygous	Compound Heterozygous	Compound Heterozygous	Homozygous	Compound Heterozygous	Compound Heterozygous	Compound Heterozygous	
ACMG classification variant 1^13^	p.(Arg714Pro): Likely pathogenic - PM1 (supporting), PM2, PP2, PP3, PP4	p.(Leu212Pro): Likely pathogenic - PM1 (supporting), PM2, PM3, PP2, PP4	p.(Leu212Pro): Likely pathogenic - PM1 (supporting), PM2, PM3, PP2, PP4	p.(Arg782Ter): Pathogenic - PVS1, PM2, PP4	c.1735+1G>A: Pathogenic - PVS1, PM2, PP4	p.(Pro50Gln) - Likely pathogenic: PM2, PM3, PP2, PP4	p.(Arg621Ter:) Pathogenic - PVS1, PM2, PP4	
ACMG classification variant 2^13^	N/A	p.(Trp559Ter): Pathogenic - PVS1, PM2, PP4	p.(Trp559Ter): Pathogenic - PVS1, PM2, PP4	N/A	p.(Arg782Ter): Pathogenic - PVS1, PM2, PP4	p.(Asp867fs): Pathogenic - PVS1, PM2, PP4	p.(Trp896Ter): Pathogenic - PVS1, PM2, PP4	
Inheritance	Biparental, consanguinity	Biparental, unrelated	Biparental, unrelated	Biparental, consanguinity	Biparental,unrelated	Biparental, unrelated	Biparental, unrelated	
Age (years)	15	8	6	7	4	2	3 months	
Sex	F	M	M	M	F	M	F	
Prenatal Growth:								
Gestation (weeks)	term	35	36	35+5	30+6	36	33+3	
Birthweight (g) [z score]	2700 [−1.4]	2030 [−1.1]	2570 [−0.36]	2190 [−0.72]	1950 [−0.59]	3100 [0.85]	2312 [1.1]	
Birth OFC (cm) [*z* score]	nk	32 [0]	32.6 [−0.11]	nk	78 [0.77]	Not recorded	34 [2.3]	
Postnatal growth :								
Weight (centile) [z score]:	0 [−3.46]	8.4 [−1.38]	4.1 [−1.73]	30 [−0.5]	0.4 [−2.69]	71 [0.55]		
Height (centile)[z score]:	nk	0.2 [−2.89]		0 [−5.1]		48 [0]		
OFC:(centile) [z score]:	2.8 [−1.91]	0.2 [−2.84]	0.5 [−2.57]	20 [−0.8]	25	97 [1.99]		
Polyhydramnios:	−	−	+	+	+	+	+	
Lethal	No	No	No	No	No	No	Yes	
*Neurology*								5 (7)
ID	Profound	Profound	Profound	Profound	Profound	Profound	profound	1 (7)
Central hypotonia	+	+	+	+	+	+	+	
Peripheral Hypotonia	+	+	+	+	+	+	+	7 (7)
Orobulbar dysfunction	+	+	+	+	+	+	+	7 (7)
Facial nerve abnormality	−	−	−	Ptosis	+	+	+	7 (7)
Vocal cord paralysis	−	+	+	+	+	−	+	6 (7)
Peripheral neuropathy	Demyelinating	Mixed	Demyelinating/dysmyelinating neuropathy	Nerve conduction studies: no motor or sensory responses	Hypomyelinating	Demyelinating	Demyelinating	4 (7)
Contractures	+	+	+	+	++	−	−	5 (7)
MRI brain	Delayed myelination, central hypomyelination	Transverse and Sagittal T2 MRI Brain images at 5 years showing lack of myelination and reduced white matter bulk and atrophic cerebellum	Lack of mylination and white matter bulk, atrophic cerebellum	Poor gyration,large cisterna magna, decreased cortical thickness wide gyri with decreased sulcation, hypomyelination of white matter, small posterior fossa	Atrophy of cerebral hemispheres and brainstem with hypomyelination	Sagittal venous sinus thrombosis at 10 days. At 4 months prominent extra axial spaces, symmetrical high signal in substantia nigra, red nuceli and superior cerebellar peduncles		7 (7)
Movement disorder	Dystonia,ataxia, tremor, head titubation	−	Dystonic spasms	−		−	−	5 (7)
Muscle/Nerve biopsy	Marked depletion of myelinated fibres, abnormally thin myelin for axon diameter	Onion bulb thinly myelinated fibres and small diameter unmyelinated fibres		Not done	Non-specific reduction in muscle fibre size	Muscle: some fibre disproportion. Mitochondrial enzyme severe complex 1 deficiency	Nerve : scattered giant axons and globally thin myelin sheaths muscle: most fibres unremarkable	
Visual impairment	+	+		+	+	+	+	2 (7)
Hearing	nk	Bilateral SNHL	Bilateral SNHL	−	−	Failed newborn check	Not known	
Other	Nystagmus	Nil	Nil					6 (7)
*Other systems*					+			3 (7)
Neonatal respiratory distress	−	+	+	+	+	+	+	
Tracheostomy	−	+	+	+	+	+	−	
GORD	+	+	+	+	+	+	+	6 (7)
PEG	−	+	+	+	+	+	−	5 (7)
Oral	Gum hyperplasia	−		Extra teeth, gum hyperplasia	−	−	Cleft palate	7 (7)
Scoliosis	Severe	+	+	+	−	+	−	5 (7)
Joint hypermobility	+	−	−	+	−	−	−	3 (7)
Hip subluxation	+	+	−	−	−	+	−	5 (7)
Other skeletal features	Swan-like finger deformity, overlapping toes, cavovarus	Pectus excavatum, deviated toes	Incomplete jaw closure	Swan neck deformity of fingers, ulnar deviation of wrists	−	Hands held with thumbs adducted and fingers flexed	−	2 (7)
*Dysmorphic features*								3 (7)
Myopathic facies	+	+	+	+	+	+	+	
Cathedral palate	+	+	+	Ridged	+	+	+	
Naevus flammus	−	−	−	+	−	−	−	7 (7)
Other		Deep plantar creases	Simple, assymetric ears, deep plantar creases	Small nails, severe eczema with overlying abnormal skin pigmentation	Anterior larynx with laryngomalacia	Resting tachycardia. Smiled socially at 12 weeks, trying to say sister's name		6 (7)
								1 (7)

Totals are shown for features seen in our cohort

### Exome library preparation and sequencing

Trio-based exome sequencing was undertaken for the five affected patients and their parents via the DDD study using the approach which has been described elsewhere [12]. A sibling pair analysis was carried out in patients 2 and 3. Their DNA samples were fragmented using the Bioruptor (Diagenode, Liège, Belgium), and indexed adaptors ligated before hybridization with the Agilent SureSelect All Exon v5 capture kit (Santa Clara, CA, USA). Paired-end 100-bp reads were sequenced on a HiSeq 2500 (Illumina, San Diego, CA, USA) to generate 75 million reads with >100× mean coverage and >95% of target bases at ≥20×. The Illumina HiSeq FASTQ sequencing reads were de-multiplexed and aligned to the reference (GRCh37/Hg19) using BWA-MEM, this is converted to BAM format file and duplicates removed using Picard. GATK (v3.4) was used for indel realignment, variant calling and quality filtering.

### Variant filtering and interpretation

Variants were annotated using Alamut-batch and a bioinformatics pipeline was designed in-house to identify shared genes where both siblings had a compound heterozygous, homozygous or X-linked recessive variant predicted to affect function. The following criteria were applied to detect rare potentially deleterious variants: minor allele frequency (MAF) > 1% in dbSNP137 variants to exclude known variants and include those with a MAF < 0.0001 (<0.01%) and 0.001 (<0.1%) in Exome aggregation consortium (ExAC http://exac.broadinstitute.org/), Exome variant server (EVS http://evs.gs.washington.edu/EVS/) or 1000 Genomes (http://www.internationalgenome.org/). Variants were restricted to non-synonymous, those affecting the conserved splice sites or those within−50/ + 10 base pairs of flanking exons predicted by Alamut-batch to affect splicing. Biparental inheritance of the *CNTNAP1* variants was confirmed by PCR/Sanger Sequencing (PCR primers available on request). Exonic variants are described according to NM_003632.2 and intronic variants according to NG_042091.1. *CNTNAP1* variants identified in this study were classified according to the American College of Medical Genetics and Genomics (ACMG) guidelines (See Supplementary Table 1 and Table 1) [13]. The data are deposited in DECIPHER and can be viewed per DECIPHER IDs provided (Patient 3 has not been deposited but the data are the same as for patient 2, his brother).

### Protein modelling

A composite, multi-template structure for CASPR was generated using the Phyre2 server (http://www.sbg.bio.ic.ac.uk/phyre2/html/page.cgi?id = index) [14] in intensive modelling mode; individual variants were then re-modeled on appropriate templates using the Swiss-Model server (http://swissmodel.expasy.org/) [15] in automated mode. All coordinate files were downloaded and visualized in PyMOL version 1.8.4.0 (Schrödinger, LLC).

## RESULTS

The clinical features and *CNTNAP1* variants in the 3 female and 4 male patients, from 6 different families, compared with the previously reported cases [3, 5–10] are presented in Table 1. All 7 patients had profound intellectual disability (ID) and a strikingly severe neurological phenotype. None of the patients had any other observed genomic aberrations.

### Perinatal

Significant polyhydramnios occurred in all but one pregnancy. This was associated with preterm labour in 5 but no patients were born at <30 weeks’ gestation. In the single pregnancy with normal liquor volume the baby was born at term. In all patients the birthweights and head circumferences were within the normal range, but 6 out of made minimal respiratory effort, were extremely hypotonic and required rapid resuscitation. Patient 7 was the most profoundly affected and following extensive investigations, died at the age of 3 months.

### Neurology, neuropathology and development

**Fig. 1:**
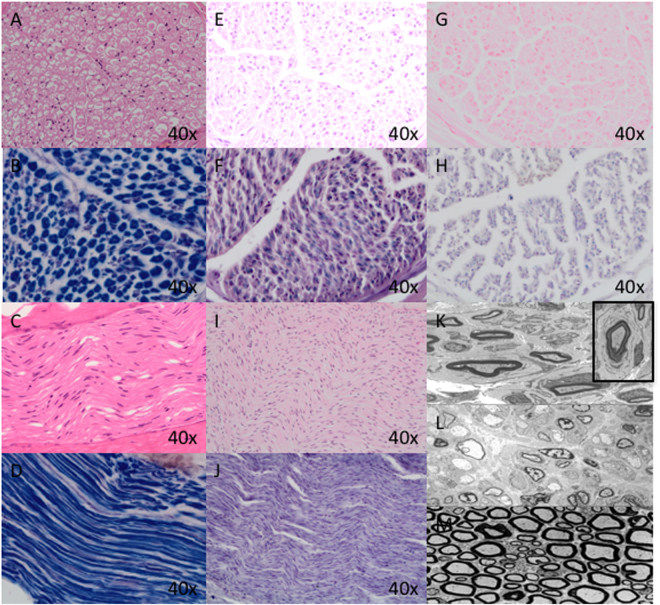
**a** Normal nerve, transverse section, H&E 40 × ; (**b**) Normal nerve, transverse section, Sol. Cyan 40 × ; (**c**) Normal nerve, longitudinal section, H&E 40 × ; (**d**) normal nerve, longitudinal section, Sol. Cyan 40 × . All normal nerve sections highlight a normal population of myelinated nerve fibres of appropriate thickness; (**e**, **f**) patient 1: Sural Nerve biopsy (TS) at 9 years of age showing marked loss of large diameter (thickly myelinated) nerve fibres, thinly myelinated fibres for axon size, and clusters of thinly myelinated fibres (axonal sprouting) on H&E (**e**) and Sol. Cyan (**f**) in keeping with hypomyelinating neuropathy; (**g**, **h**) patient 2: Sural Nerve biopsy (TS) at 10 months of age showing modest loss of large diameter (thickly myelinated) fibres, presence of thinly myelinated fibres, clusters of thinly myelinated fibres and a rare onion bulb on H&E (**g**) and Sol.Cyan (**h**) consistent with hypomyelination; (**i**, **j**) Patient 7: Sural Nerve biopsy (LS) following death at 3 months of age (autopsy specimen). Demonstrates widespread, almost complete loss of myelinated fibres on H&E (**i**) and Sol. Cyan (**j**) with a few residual thinly myelinated fibres. **k** Patient 2: Electron microscopy (EM) highlighting clusters of thinly myelinated fibres with only a few residual appropriately myelinated fibres. Inset highlights single onion bulb in keeping with active demyelination. **l**. Patient 7: EM highlighting extensive loss of myelinated nerve fibres with a residual rare thinly myelinated fibre. **m** Electron Microscopy of a normal nerve showing a normal complement of large myelinated fibres and thinly myelinated fibres with proportionate axons

All cases had both central and peripheral hypotonia, with variably demyelinating/hypomyelinating neuropathy and variable additional neurological signs: patient 1 had nystagmus, ataxia, tremor, head titubation and 2 other cases had dystonia. MRI brains scans consistently showed hypomyelination/demyelination, variably reduced white matter bulk and cerebral atrophy. The results of the nerve biopsies for patients 1,2,3,4 are shown in Fig. 1. The muscle biopsies were non-specific and did not show any neurogenic pathology despite the findings of hypomyelination in the nerve biopsies. This might reflect the time course, as many of the muscle biopsies were taken early on during the disease, and in most cases, a substantial time prior to the nerve biopsies. All our patients had severely delayed development except one. He had a social smile at the age of 12 weeks and is attempting single words at the age of 2. Patient 1, the oldest in the cohort, can communicate some preferences through noise and body language and is described as enjoying certain television programmes.

### Respiratory

Six out of 7 patients had severe respiratory distress at birth, 5 requiring tracheostomy. In the sixth case, a tracheostomy was not considered appropriate as the prognosis was poor. In patients 2 and 3, the tracheostomies were successfully decannulated, with both boys requiring overnight non-invasive ventilation. However, both boys have subsequently required re-insertion of their tracheostomies due to issues with repeated respiratory infections and respiratory compromise, which have resulted in multiple intensive care/high dependency unit admissions.

### Dysmorphology

**Fig. 2:**
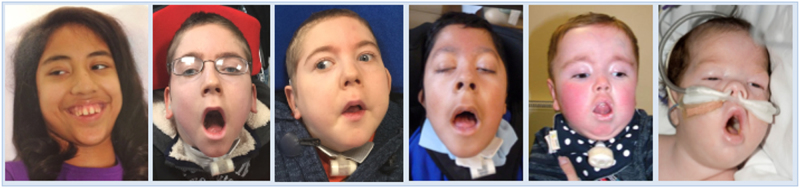
Facial features of patients 1,2,3,4,6,7 from left to right. Patient 1 is reported with the mildest phenotype, which is apparent from the photo comparisons and makes her appear more atypical. Note the consistent narrow down-slanting palpebral fissures, full rounded eyebrows, myopathic facies and mouth held wide open. Tracheostomy is shown in patients 2,3, 4 and 6

The facial features of the patients are depicted in Fig. 2. All patients have consistent myopathic facial features (Fig. 2) and one a naevus flammus.

### Other features

Gum hyperplasia is seen in 2 patients, one of whom had extra teeth. All patients had a typical ‘cathedral’ palate, but only patient 7 had a cleft. An anterior larynx with laryngomalacia was reported in patient 5. Patient 6 has a resting tachycardia of unknown cause. Patient 4 has small nails, and severe eczema with overlying abnormal skin pigmentation.

### Variants

**Fig. 3:**
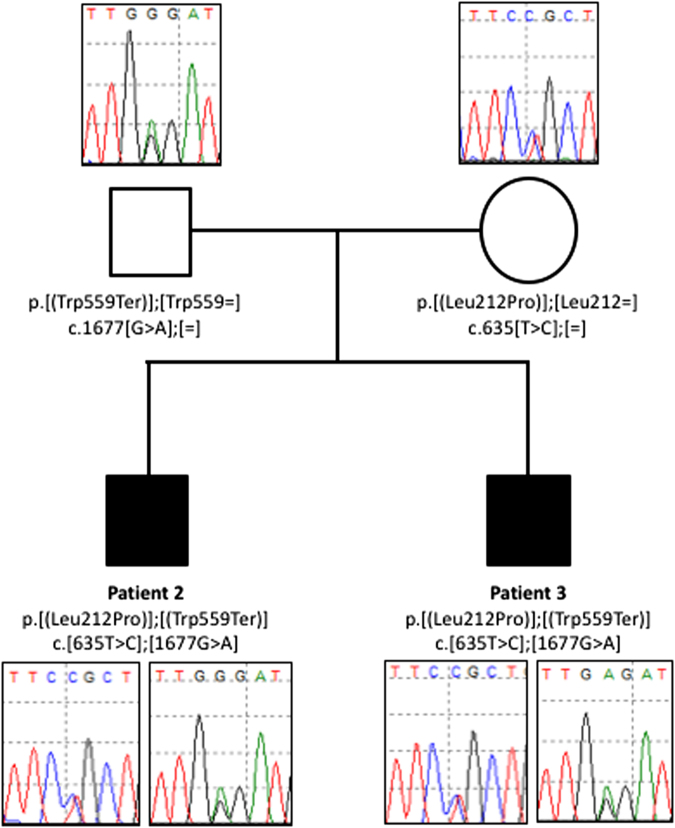
Sanger sequencing of variants identified in *CNTNAP1* in the affected brothers (patients 2 and 3) and their parents. Open symbols: unaffected; filled symbols: affected; square symbols: male and circular symbols: female The nucleotide and amino acid changes are indicated

**Fig. 4:**
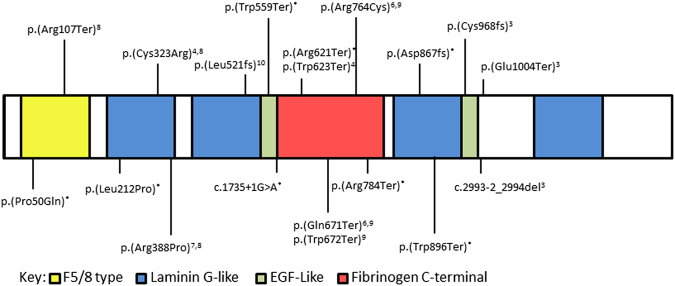
Representation of the CASPR protein showing functional domains and approximate location of all reported variants to date. Those marked with an * were identified in this study, others are marked with the paper reference. Variants are described according to NM_003632.2, NG_042091.1

We identified 9 novel *CNTNAP1* variants that are predicted to affect CNTNAP1 function in 6 families by whole-exome sequencing (Table 1). These included three missense variants, four nonsense variants, one frameshift variant and one splice site variant. Two patients from consanguineous families were homozygous for a novel missense (c.2141 G > C, p.(Arg714Pro)—patient 1)) and a nonsense variant (c.2344 C > T, p.(Arg782Ter)–patient 4. In each family, the parents were heterozygous carriers of the compound heterozygous or homozygous variants identified in the affected proband. The other 5 patients were compound heterozygotes for either two protein truncating variants (patients 5 and 7) or a missense and nonsense variant (patients 2 and 3, Fig. 3) or a frameshift and missense variant (patient 6). One of the nonsense variants, c.2344 C > T p.(Arg782Ter), was identified in two families. All variants described in this study and those previously reported to affect function are shown in Fig. 4. The three novel missense variants were predicted to be affecting function according to the ACMG guidelines (Table 1, and Supplementary Table 1).

### Protein modelling

**Fig. 5:**
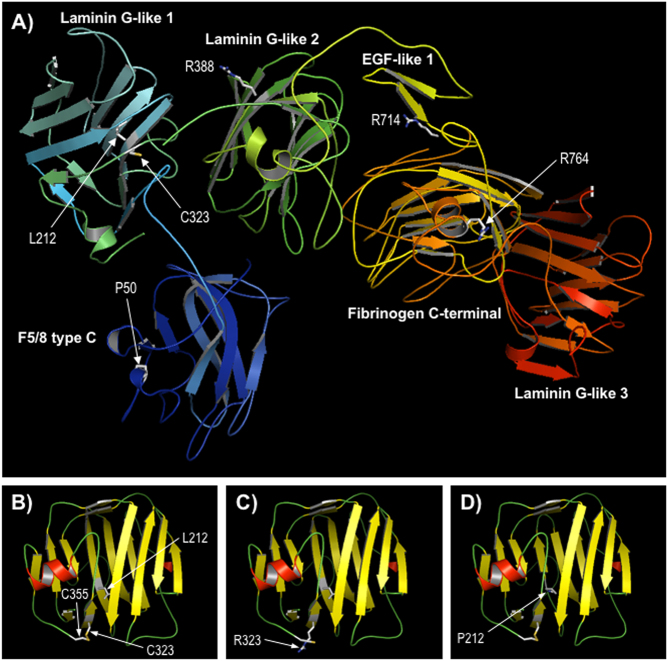
Comparative modelling of CASPR. **a** Predicted structure of CASPR residues 21–960. The sequence of CASPR (residues 1–1384) was modeled using the Phyre2 server; a multi-template, high-confidence model (~98% of residues modeled at > 90% confidence) was obtained for a contiguous region spanning residues 21–960, which includes most of the extracellular domain (1–1284) and includes all missense variants discussed here. The protein is shown in ribbon format coloured by secondary structure succession (N-terminal, blue to C-terminal, red); the sidechains of positions of missense variants are shown in stick format and labelled; domain annotation is taken from the InterPro database entry for CASPR (http://www.ebi.ac.uk/interpro/protein/P78357). **b**–**d** Modelling of the Laminin G-like 1 domain only (residues 174–355), based on template 3poyA, for wild-type CASPR and variants p.Cys323Arg and p.Leu212Pro respectively; protein is shown in ribbon format, coloured by secondary structure type (red, α-helix; yellow, β-strand; green, loop); the disulphide bond between cysteines 323 and 355 in B is shown by a yellow line; view is rotated compared to Fig. 4a for clarity

Since there are no experimental structures for any regions of CASPR, a 3-dimensional structure of the protein was predicted using the Phyre2 protein modelling server (Fig. 5). A high confidence model was obtained for a contiguous region covering residues 21–960 of CASPR, which includes all novel missense variants reported here or previously identified as affecting function (Fig. 5a). All substitutions lie within conserved structural and functional domains and thus could potentially cause deleterious effects on CASPR function. To examine the effects of specific variants, template structures used by Phyre2 for calculation of the composite model were used to re-model wild-type and variant sequences over the appropriate regions. Modelling suggested that all missense variants were likely to affect the underlying structure of CASPR, to a greater or lesser extent, which is consistent with loss of protein function. For example, the p.(Leu212Pro) and p.(Cys323Arg) variants both lie in the first Laminin G-like domain of CASPR (residues 203–355). Predictive modelling showed that p.Cys323 forms a disulphide bond to p.Cys355, stabilizing the loop region between this domain and the following Laminin G-like domain 2 (Fig. 5b). This bond is disrupted by the p.(Cys323Arg) variant, likely leading to destabilization and impaired folding of the protein in this region (Fig. 5c). Similarly, the p.(Leu212Pro) substitution was predicted to interrupt a region of β-strand (Fig. 5d), which is also likely to result in impaired folding of this domain. The p.(Arg764Cys) variant, which lies in the core of the Fibrinogen C-terminal domain of CASPR, was also predicted to have a strong effect on the underlying domain structure, while variants p.(Pro50Gln), p.(Arg388Pro) and p.(Arg714Pro) either occur in flexible loop regions or were expected to have lesser impact on the domain structure (data not shown). The predicted effects of all variants are summarized in Supplementary Table 2.

## Discussion

This series of patients extends the phenotype of *CNTNAP1-*related CHN and indicates that survival into infancy and childhood can occur in this condition. By contrast, of the 19 (the number of individuals refers to the number of individuals who have been specifically reported in the literature. Several case reports refer to further siblings or family members with either very limited data and/or no testing and we have not included them in this total count) previously published cases, only 6 survived infancy. Vanderver et al. reported 2 patients who were 6 years old, although both had a sibling who died of respiratory compromise. Hengel et al. very recently reported a family with 3 children with a homozygous truncating variant, all of whom are alive. Lakhani et al. likewise reported a family in which one child remains alive at age 13 “in a persistent vegetative state” although two affected siblings died within the first hour of life. Amongst our cohort, only one child followed similar early demise compared to the majority of those reported. Furthermore, one of our patients had no neonatal respiratory distress and experienced few respiratory problems by her current age of 15 years, demonstrating the potenially broad phenotypic spectrum associated with CNTNAP1 variants. This discrepancy may reflect differences in the ascertainment of the children—the majority of the cases in our study were diagnosed through a WES looking for diagnoses in children with developmental delay, which focussed (albeit not exclusively) on living children. This proves that the spectrum of disease is broader than previously thought.

While all previously described patients and most of ours had marked ID, we recognised that two of our patients had made some progress, so developmental delay in *CNTNAP1-*related CHM may not always be profound. One of our patients had a cleft palate and Vanderver et al [8] reported a single patient with a submucosal cleft palate. Hengel et al. have now also reported this feature in two of their patients and so this may represent a less frequent phenotypic association with *CNTNAP1-*CHN (4/26). We also noted thickened gums in two of our patients and this has also been reported in one patient by Hengel et al., again possibly representing a less frequently reported association, although it is possible this was also not specifically looked for or noted in previous reports.

Early-onset hereditary neuropathies can be described in terms of age of onset and hence divided in to CHN and Dejerine–Sottas neuropathy starting in infancy [16]. De novo dominant variants predicted to affect function have been well reported in association with early-onset neuropathies in *PMP22*, *MPZ* [16–19] and *EGR2* [20, 21,]. In addition, variants in other Hereditary Motor and Sensory Neuropathy genes have been associated with overlapping phenotypes. Baets et al. [16] performed direct sequencing of the coding regions of *MFN2, PMP22, MPZ, EGR2, GDAP1, NEFL, FGD4, MTMR2, PRX, SBF2* and *SH3TC2* in 77 isolated cases of hereditary neuropathy starting in the first year of life. They detected variants predicted to affect function in 35 patients but only three are described as having respiratory insufficiency. One, who first came to medical attention due to delayed motor milestones, had a variant in *SH3TC2*, but had less marked hypotonia and was able to walk at 24 months. Another with congenital hypotonia and respiratory problems in the neonatal period, had a variant in *MPZ* However the clinical picture appeared milder than in our cohort: the patient was able to walk with support until the age of 6 years, after which he was wheelchair bound. *MPZ* has been reported in association with a CHN picture, but usually without the respiratory problems by us. [16–19] Furthermore, while the symptoms are of early onset, the weakness is not as severe and walking has been achieved by patients. Unlike in *CNTNAP1*-CHN, cranial nerve involvement is not reported in association with variants in *MPZ*. The third patient had a variant in *EGR2* but was able to walk at the age of 3, and is therefore distinct from patients in our cohort. Variants in *EGR2* [20, 21,] have previously been reported in association with severe CHN and compromise of cranial nerve function. None of the patients with variants in these 3 genes have had the severe respiratory insufficiency from birth as we describe. Additionally, patients with variants in *EGR2* can have normal intellect-a clear difference from the phenotype of *CNTNAP1*-CHN. Polyhydramnios and associated premature labour, does not appear to be a feature in any of these other groups and therefore provides a clue in the severe neonatal presentation of CHN with respiratory insufficiency. Hearing and visual impairment are not a widely reported finding in CHN in association with any of these other genes and again this appears to be a specific finding in *CNTNAP1*-CHN although it is not seen in all patients.

Comparative modelling of the missense variants suggests a possible genotype–phenotype relationship, in that those substitutions which are expected to have the least effect on protein structure seem to correlate with a milder phenotype in our patients. Patient 1, who has a *CNTNAP1* homozygous missense variant (c.2141[G > C];[G > C], p.[(Arg714Pro)];[(Arg714Pro)], has no respiratory problems, which is striking compared to the rest of the group. Protein modelling has shown that while the variant may disrupt ligand interactions, the underlying secondary structure of the protein and structural core remain unchanged. This could account for the respiratory and other phenotypic differences. Patient 6 appears to be making the most progress developmentally (c.[149 C > A];[c.2600del], p.[(Pro50Gln)];[(Asp867fs)]). The p.(Pro50Gln) variant is modelled to cause a possible altered topology of local loop with low impact. This hypomorphic variant may be partially ameliorating the phenotype in this child. In comparison, our only patient who died at few months of age, had compound heterozygous nonsense variants. We therefore speculate that there may be a genotype–phenotype relationship in which hypomorphic missense variants modify the deleterious effect on peripheral/central myelination and development of paranodal junctions. However, we acknowledge that, at present, there are only a very limited number of known affecting function missense variants in *CNTNAP1* and the role of various domains in CASPR in ligand binding is still poorly understood. Investigation of further patients with *CNTNAP1*-CHN and functional assays to assess the effects on protein structure/function will elucidate this relationship. There does not appear to be a clear molecular mechanism to account for the CHN presentation vs. the AMC presentation. It is noteworthy as well that Hengel et al. reported a family of three affected siblings, in whom two siblings had AMC and the third did not. It is possible that these differences are more to do with diagnostic labelling and that this all represents a spectrum of *CNTNAP1* related disease.

## Conclusion

Our study, in combination with previous reports, demonstrates that biallelic variants in *CNTNAP1* cause a distinct recognisable syndrome of polyhydramnios, severe congenital hypomyelinating neuropathy with central hypotonia and cranial nerve involvement, severe respiratory insufficiency often necessitating tracheostomy, profound developmental delay/intellectual disability. Hearing and visual impairment may be a significant further clue to this diagnosis. In contrast with previous reports, a significant number of patients with *CNTNAP1*-CHN may survive infancy into childhood. Hypomorphic missense variants may influence the severity of the phenotype, resulting in a less severe picture. Further cases will confirm the full clinical spectrum of this disorder and genetic/functional studies will clarify and extend the implicatons of our protein modelling. Neonates presenting with this phenotype will benefit from early genetic definition to inform clinical management and enable essential genetic counselling for their families.

## Electronic supplementary material

Supplementary Table 1

Supplementary Table 2

Supplementary Table 3
