# Supplementary material for: Phenotype of CNTNAP1: a study of patients demonstrating a specific severe congenital hypomyelinating neuropathy with survival beyond infancy
Source: Eur J Hum Genet. 2018 Mar 6;26(6):796–807. doi: 10.1038/s41431-018-0110-x (PMC5974240; doi:10.1038/s41431-018-0110-x)
Supplement: Supplementary file 2 — Supplementary Table 2 [file 41431_2018_110_MOESM2_ESM.docx]

| **Variant** | **CASPR domain** | **template** | **Position within domain** | **Effect on modelled structure** | **Likely outcome and impact on structure/function** |
| --- | --- | --- | --- | --- | --- |
| Pro50Gln | F5/8 type C | 4bsxV | occurs in extended loop at domain surface; possibly stabilizes loop due to rotational constraint of proline | none apparent in models; possible destabilization of loop region | possible altered topology of local loop; low impact |
| Leu212Pro | Laminin G-like 1 | 3poyA | within β-strand; sidechain buried in hydrophobic core of domain | disruption of secondary structure | misfolding, loss of function; high impact |
| Cys323Arg | Laminin G-like 1 | 3poyA | within β-strand; forms disulphide bond with Cys355 in loop between Laminin G-like domains 1 and 2 | loss of disulphide bond with Cys355 | misfolding, loss of function; high impact |
| Arg388Pro | Laminin G-like 2 | 3poyA | within β-strand; sidechain exposed to surface, possibly for ligand interaction | loss of surface charge; no apparent change to secondary structure | unknown - potential to disrupt ligand binding |
| Arg714Pro | Fibrinogen C-terminal | 4aejA | occurs in extended loop at domain surface; may stabilize loop folding by hydrogen bonding to other residues within loop | altered topology in loop (low confidence modelling) and loss of surface charge; loss of hydrogen bonding within loop | unknown - potential to disrupt ligand binding |
| Arg764Cys | Fibrinogen C-terminal | 4aejA | occurs in turn between anti-parallel β-strands; probably stabilizes turn and alignment of strands by hydrogen bonding to residues in neighbouring turns | loss of hydrogen bonding to residues in neighbouring turns, some changes to extent of β-strands; possible disruption of inter-strand disulphide bond Cys677-Cys732 | probable misfolding of domain, loss of function; high impact |

**Supplementary table 2. Summary of comparative modelling of CASPR missense variants**. Individual domains of CASPR were modelled for wild-type and variant sequences using the most appropriate structural template for that region, as identified in results of Phyre2 multi-template modeling. Modelling of individual domains was performed using the SWISS-MODEL server in automated mode, with user-specified templates as indicated in the table; super-imposition of wild-type models on the composite, multi-template model indicated strong concordance between results from the two modelling servers (data not shown).
